# Exploring the potential of computer vision analysis of pupae size dimorphism for adaptive sex sorting systems of various vector mosquito species

**DOI:** 10.1186/s13071-018-3221-x

**Published:** 2018-12-24

**Authors:** Mario Zacarés, Gustavo Salvador-Herranz, David Almenar, Carles Tur, Rafael Argilés, Kostas Bourtzis, Hervé Bossin, Ignacio Pla

**Affiliations:** 1 Departamento de Ciencias Experimentales y Matemáticas, Universidad Católica de Valencia “San Vicente Mártir”, C/Guillem de Castro 94, 46003 Valencia, Spain; 2 Departamento de Expresión Gráfica, Proyectos y Urbanismo, Universidad CEU Cardenal Herrera, Valencia, Spain; 3 Grupo Tragsa, Avda. de la Industria 26, 46980 Paterna, Valencia Spain; 4 Insect Pest Control Section, Joint FAO/IAEA Division of Nuclear Techniques in Food and Agriculture, Wagramerstrasse 5, PO Box 100, A-1400 Vienna, Austria; 5 Laboratoire d’Entomologie Médicale, Institut Louis Malardé, BP 30, 98713 Papeete, Tahiti French Polynesia; 6 IRD, AP-HM, SSA, VITROME, IHU-Méditerranée infection, Univ. Aix Marseille, Marseille, France

**Keywords:** Sterile insect technique, Biometrical analysis, Morphometrics frequency distribution models, Sexual size dimorphism, Sex sorting methods, *Aedes aegypti*, *Aedes albopictus*, *Aedes polynesiensis*, *Anopheles arabiensis*

## Abstract

**Background:**

Several mosquito population suppression strategies based on the rearing and release of sterile males have provided promising results. However, the lack of an efficient male selection method has hampered the expansion of these approaches into large-scale operational programmes. Currently, most of these programmes targeting *Aedes* mosquitoes rely on sorting methods based on the sexual size dimorphism (SSD) at the pupal stage. The currently available sorting methods have not been developed based on biometric analysis, and there is therefore potential for improvement. We applied an automated pupal size estimator developed by Grupo Tragsa with laboratory samples of *Anopheles arabiensis, Aedes albopictus*, *Ae. polynesiensis*, and three strains of *Ae. aegypti*. The frequency distribution of the pupal size was analyzed. We propose a general model for the analysis of the frequency distribution of mosquito pupae in the context of SSD-sorting methods, which is based on a Gaussian mixture distribution functions, thus making possible the analysis of performance (% males recovery) and purity (% males on the sorted sample).

**Results:**

For the three *Aedes* species, the distribution of the pupae size can be modeled by a mixture of two Gaussian distribution functions and the proposed model fitted the experimental data. For a given population, each size threshold is linked to a specific outcome of male recovery. Two dimensionless parameters that measure the suitability for SSD-based sorting of a specific batch of pupae are provided. The optimal sorting results are predicted for the highest values of SSD and lowest values of intra-batch variance. Rearing conditions have a strong influence in the performance of the SSD-sorting methods and non-standard rearing can lead to increase pupae size heterogeneity.

**Conclusions:**

Sex sorting of pupae based on size dimorphism can be achieved with a high performance (% males recovery) and a reasonably high purity (% males on the sorted sample) for the different *Aedes* species and strains. The purity and performance of a sex sorting operation in the tested *Aedes* species are linked parameters whose relation can be modeled. The conclusions of this analysis are applicable to all the existing SSD-sorting methods. The efficiency of the SSD-sorting methods can be improved by reducing the heterogeneity of pupae size within rearing containers. The heterogeneity between batches does not strongly affect the quality of the sex sorting, as long as a specific separation threshold is not pre-set before the sorting process. For new developments, we recommend using adaptive and precise threshold selection methods applied individually to each batch or to a mix of batches. Adaptive and precise thresholds will allow the sex-sorting of mixed batches in operational conditions maintaining the target purity at the cost of a reduction in performance. We also recommend a strategy whereby an acceptable level of purity is pre-selected and remains constant across the different batches of pupae while the performance varies from batch to batch to fit with the desired purity.

## Background

There is a global renewed interest in area-wide integrated mosquito management strategies based on the mass production and release of sterile males to suppress target populations [1–4]. These techniques are usually referred to as genetic control methods and include, among others, the sterile insect technique (SIT), the incompatible insect technique (IIT) and the release of insects carrying a dominant lethal gene (RIDL) [1, 4–7]. Several small-scale projects have demonstrated the high potential of these strategies to suppress mosquito populations [6, 8–10]. The scaling-up of these projects from pilot to operational has been hampered by several problems, the most significant one being the lack of an efficient sex-sorting method [1, 11, 12]. Given that only the female mosquitoes bite and transmit the human pathogens, those methods must be capable of ensuring a predefined acceptable level of female contamination while maximizing the male pupae recovery.

The successful use of genetic sexing strains (GSS) for the sex sorting of *Ceratitis capitata* and other fruit fly species [13–17] has encouraged researchers to develop similar GSS strains for mosquitoes. GSS strains that can be sex-sorted at early developmental stages (eggs or L1) are generally accepted to be the optimal solution for mass-scale SIT and related techniques [11, 12]. A genetic sexing strain based on the tolerance to dieldrin has been developed for *Anopheles arabiensis*; however, this strain presents several problems and has limited potential for SIT applications [18, 19]. In addition, several transgenic genetic sexing strains developed for different mosquito vector species are also of limited applied potential due to either lack of stability, low male performance or subject to extensive regulation [11, 12].

The current lack of a functional GSS has led to the mosquito population suppression projects to use alternative ways in the sex-sorting process. For the mosquito species with strong sexual size dimorphism (SSD), mainly *Aedes* and *Culex* species, mechanical methods have been generally adopted for sorting [6, 8, 20]. Although several designs and proposals for sex sorting on a mass scale have been suggested in the past [21, 22], all mosquito genetic control programmes currently use either plate separators [23] or sieves [8] for sex sorting that have been devised for small-scale rearing conditions. The development of new designs with automation capability for unattended sorting would increase the efficiency of those projects and allow their upgrade to large operational programmes [12, 24].

The efficiency of SSD-sorting methods in terms of male recovery, female contamination and speed depends on technical and biological factors. The technical features basically affect the rate of separation per time unit, and differ between methods. The main biological determinant is the size distribution between sexes and their overlap as well as the effect of rearing conditions on this characteristic. All the SSD-sorting methods rely on the same principle: the separation in two samples by a threshold size. It should be noted that an analysis of the biological determinants of the distribution of size will in principle be applicable to all SSD-sorting methods

In order to improve the performance of new designs of sex-sorting methods based on SSD, a previous biometric analysis is required, specifically dealing with the analysis of the frequency distribution of the size of sexes. However, there is scarce information regarding the distribution of size in mosquitoes. Usually, the scope of the biometric studies in mosquitoes has been to find correlations between the body size and other biological traits [25–29], providing only point and variance estimates, and only a limited number of studies have included detailed frequency distributions [30–32]. For insects, most of the frequency distributions of the size can fit to normal probability functions. When a strong SSD is present, each sex can fit to an independent normal curve [33]. SSD is generally assumed as a species-specific (or population-specific) trait with a narrow degree of variation caused by complex interactions of factors [34–37].

Several experiments have shown that variations in the mosquito larval rearing conditions can increase or reduce the average size of the resulting pupae [38–41]. The SSD is slightly influenced by intraspecific competition [40, 42], but not by food availability [38] or the pollution by conspecifics [43] as size of both sexes is equally affected by these parameters and the difference between the average size of each sex remains constant. The objective of the present study is to optimize the utilization of the SSD-sorting methods through the understanding of the frequency distribution in the pupal size of different mosquito species and strains, with the ultimate goal to: (i) understand the performance of the current sex-sorting methods in different conditions; (ii) assess the relationship between the parameters of importance for sex-sorting devices: female contamination and male recovery; (iii) evaluate the suitability of size-based sex sorting methods for different species and strains of mosquitoes; and (iv) propose features that will optimize the performance of SSD-sorting methods.

To achieve these objectives, we developed a general model for the size distribution of mosquito at the pupal stage. The frequency distribution can be modelled as a mixture of two normal probability density functions. We analyzed the frequency distribution in size of four important mosquito vector species that are currently the target of area-wide integrated vector control projects using SIT-based methods: *Aedes aegypti*, *Ae. albopictus*, *Ae. polynesiensis* and *Anopheles arabiensis*. The intraspecific variation is also evaluated for *Ae. aegypti*, since three different laboratory strains were included in the analysis. The use of an automated pupae size estimator system based on artificial vision developed by Grupo Tragsa, Spain, allowed the collection of a large amount of size measurements thus facilitating the achievement of our objective.

## Methods

### Laboratory strains

The *Aedes aegypti* Sri Lanka strain originates from mosquitoes collected from the Narahenpita area, District of Colombo, Western Province, Sri Lanka. This strain was kindly provided by Ms. Asha Wijegunawardana (University of Kelaniya, Sri Lanka) and has been maintained in the Insect Pest Control Laboratory of the Joint Food and Agriculture Organization and International Atomic Energy Agency (IPCL-Joint FAO/IAEA) laboratories since 2017. F_28_ mosquitoes from this strain were analyzed in the present study.

The *Ae. aegypti* GSS has been developed by classical genetic approaches and has been maintained in the IPCL-Joint FAO/IAEA since 2017. F_5_ mosquitoes from this strain were used in the present study.

The *Ae. aegypti* WB2 line was recently generated by transfer of *Wolbachia w*AlbB from *Aedes albopictus* into *Aedes aegypti* via embryonic microinjection at Michigan State University (personal communication, Zhiyong Xi), and has been maintained in the IPCL laboratories since 2016. This strain was introgressed into the genomic background of an *Ae. aegypti* strain from Brazil, provided by Professor Margareth Capurro (University of Sao Paolo, Brazil), through a series of seven backcrosses using in every generation *Wolbachia*-infected females mated with *Ae. aegypti* Brazil males. This resulted in the construction of the *Ae. aegypti* WB2-BRA strain used. F_12_ mosquitoes from this strain were analyzed in the present study.

The incompatible *Ae. polynesiensis* “Aito” (BC9) strain carries *Wolbachia* B from *Ae. riversi*. This strain was generated through multiple backcrosses between *Aedes riversi* females and *Aedes polynesiensis* aposymbiotic males (Hapairai, 2013). This strain which has been maintained at Institut Louis Malardè (ILM), Tahiti since 2010 was recently used in a pilot IIT field study on the atoll of Tetiaroa, French Polynesia (Bossin et al. manuscript in preparation).

The *Ae. albopictus* Rimini strain was originated from field collections in northern Italy. It has been maintained in the IPCL since 2010.

The *An. arabiensis* Dongola strain was originated from the Northern State of Sudan. It has been maintained in the IPCL since 2005. It is also available at the Malaria Research and Reference Reagent Resource Center, MR4, as MRA-856.

### Mosquito rearing

Standard rearing conditions have been used for the maintenance of experimental colonies, egg collection and hatching of *Ae. albopictus* and *Ae. aegypti* [44, 45], *Ae. polynesiensis* [39], and *An. arabiensis* [46, 47] colonies.

### Pupae production

For each species or strain, three larval containers were prepared for pupae production as described below. These three replicates represented a random sample of the different rearing units found in a mass rearing facility.

For *Ae. aegypti* and *Ae. albopictus*, 2000 first-instar larvae were introduced in white acrylonitrile-butadiene-styrene (ABS) plastic trays (41 × 30 × 8 cm) with 1.5 l of deionized water. Since larvae of *Ae. polynesiensis* must be reared under lower densities [39], 1200 first-instar larvae were introduced in 40 × 60 × 15 cm containers with 4 l of water. The larvae were fed with the standard *Aedes* IPCL diet [48, 49] at a concentration of 75 g per liter of diet. The diet regime ranged from 0.2 mg of dry weight per larvae on the first day to 0.8 mg on the last days.

Different batches of eggs of *Ae. arabiensis* were hatched in white plastic trays (41 × 30 × 8 cm). Two days after the hatching, approximately 500-1000 larvae were visually isolated in the same kind of trays with 1.5 l of deionized water. The larvae were fed with the standard *Anopheles* IPCL diet [46], in a concentration of 10 g/l ranging from 5ml on the first day to 20 ml on the last days.

All pupae in each container were collected on a daily basis starting at 24 hours from the beginning of the pupation. A batch of pupae was defined as all the pupae produced in 24 hours for a specific species/strain and replicate(s). The selected batches were sex-sorted under a binocular microscope. All the pupae in a batch were classified into males and females groups, and the resulting samples are referred to as batch-sex groups. The batch where the proportion of male and female was closer to 50%, usually on the 2nd or 3rd day from the beginning of pupation, was selected for the analysis.

### Measurement of pupal size

**Fig. 1:**
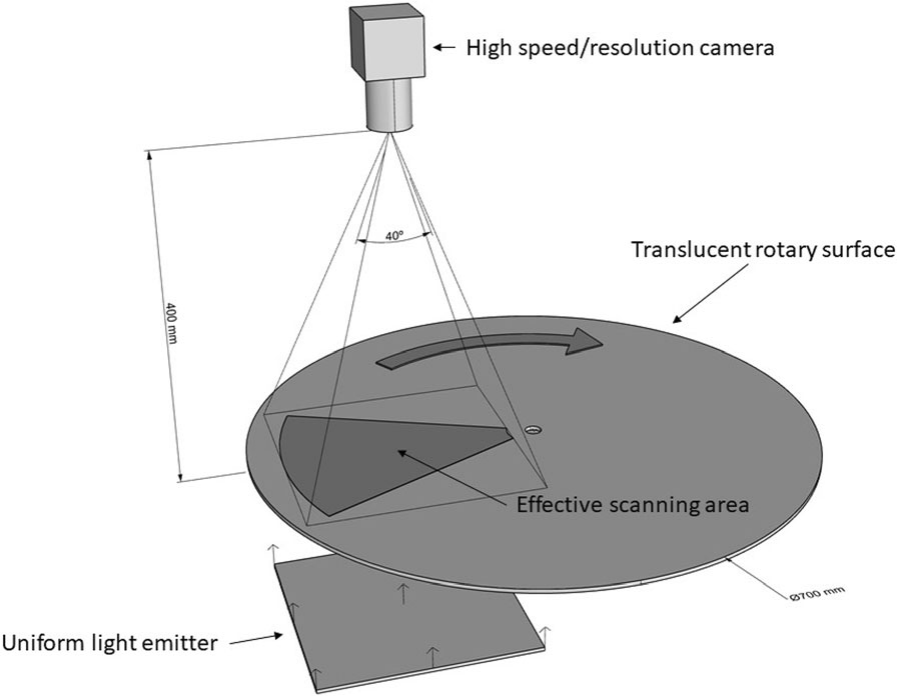
Continuous image capture size

The lateral profile area of the pupae was automatically measured by means of a computer vision system (Fig. 1), which comprises: (i) a translucent rigid surface with circular uniform movement, acting as conveyor on which the mosquito pupae are arranged; (ii) a uniform and high intensity led white light backlight system and (iii) a high resolution/high speed camera placed in top position. In this way, the mosquito pupae pass continuously under the camera while being backlit by the lighting system. The backlighting of the pupae allows photograph of them with a high contrast, which facilitates their subsequent extraction and isolation from the background (segmentation). In order to increase the precision and accuracy of the measurement of the areas, avoiding errors due to the effects of refraction of light by water droplets, the size of the pupae was measured in dry conditions for each session.

A factor of special importance is that the backlight system must guarantee an illuminated area with a uniform intensity, at least in the interest area of capture of the camera. This is because the light intensity directly affects the size of the areas extracted in the segmentation process, and variations in the intensity could result in errors in the relative measurements. However, even with a uniform backlighting system, minor errors may occur in the measurement due to the position of each pupa with respect to the position of the camera (different projected areas) and electrical noise in the silicone sensor of the camera. To minimise these phenomena, several pictures of each pupa are recorded while in the camera capture area (around 15 shots per pupa), and then the median of all the measurements of each individual is chosen as the value of size. So, each individual has to be identified and its path has to be tracked. For this task, we have developed a predictive tracking algorithm, based on Kalman filters [50], which is able to identify and track individuals in their rotational displacement under the field of view of the camera. Additionally, the algorithm is robust enough to follow the characteristic rapid movements of the pupae in dry conditions, which are quite active.

The median size in square pixels is then transformed to a unidimensional parameter by the square root to linearize the measure of size. The measure of size presented in this study is then the square root of the pixel area. The outcome of the analysis is scale-independent, and the conclusions are valid regardless of the actual value of this magnitude. Since the pupae were sex sorted manually, we assume that a certain degree of identification error occured. Errors in the manual sex identification can affect the estimation of the statistical parameters, especially those pupae with size far larger or smaller than the corresponding sex average value. In order to minimize this effect, we considered each value that exceeded two standard deviations from the average as an error in sex identification. These values were subsequently excluded from the analysis.

### Model for the frequency distribution of size

The proposed model relies on the basic assumption that the probability density function for the pupal size of each sex follows a Gaussian distribution, with the mean and standard deviation as the characteristic parameters. The mixture distribution for this situation is:

1 $$ f(x)={\alpha}_m\mathcal{N}\left(x;{\mu}_m,{\sigma}_m\right)+{\alpha}_f\mathcal{N}\left(x;{\mu}_f,{\sigma}_f\right) $$

where N(x; μ_i_, σ_i_) is the normal probability density function for size (x), with mean μ_i_ and standard deviation σ_i_. The scalars α_i_ are the proportions of each sex in the model, being α_m_ + α_f_ = 1. The subscripts m and f denote males and females respectively.

### Predictions from the model

One of the goals of our model is to provide a statistical tool to estimate the theoretical outcomes of male recovery and female contamination. In order to quantify them we introduce the performance (*PER*) and purity (*PUR*) functions defined as follows:

2 $$ PER(x)=\frac{recovered\ males\ from\ the\ original\ sample\ for\ a\  given\ x}{males\ in\ the\ original\ sample} \times 100 $$

3 $$ PUR(x)=\frac{recovered\ males\ from\ the\ original\ sample\ for\ a\  given\ x}{recovered\ pupae\ from\ the\ original\ sample\ for\ a\  given\ x} \times 100 $$

**Fig. 2:**
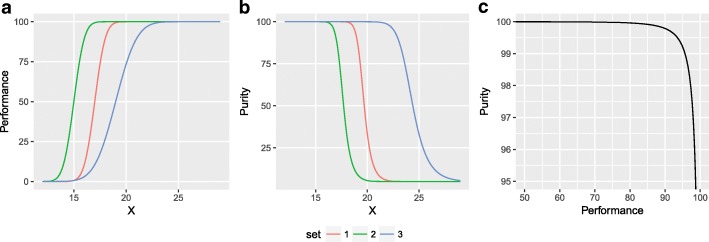
Depiction of the purity-performance relationship under a mixture of two Gaussian distributions applied to the analysis of sex sorting by size. The graphs consider α_m_ = 0.5 and three different sets of parameters s = {μ_m_, σ_m_, μ_f_, σ_f_}, s1 ={10, 1, 11, 1}, s2 = {8, 1, 9, 1} and s3 = {12, 2, 14, 2}. The performance of sets 2 and 3 is obtained by translating and scaling the performance of set 1: PER2(x) = PER1(x + 2), PER3(x) = PER1(x−2/2). The same transformations are applied to purity functions. **a** Purity *versus* size (X). **b** Performance *versus* size (X). **c** Purity versus performance

For any given size threshold (*x*), *PER* provides the male recovery percentage and *PUR* the percentage of males on the sorted sample. Assuming the model given by Equation 1, it is easy to show that both functions can be estimated in terms of the normal distribution function:

4 $$ PER(x)=\varPhi \left(\frac{x-{\mu}_m}{\sigma_m}\right)\times 100 $$

5 $$ PUR(x)=\frac{\alpha_m\Phi \left(\frac{x-{\mu}_m}{\sigma_m}\right)}{\alpha_m\Phi \left(\frac{x-{\mu}_m}{\sigma_m}\right)+\left(1-{\alpha}_m\right)\Phi \left(\frac{x-{\mu}_f}{\sigma_f}\right)}\times 100 $$

where Φ denotes the standard normal cumulative distribution function. In order to determine both functions, the parameters {α_m_, μ_i_, σ_i_} of the model must be known. Purity and performance are inversely linked. A decrease in female contamination can be achieved by reducing the value of the threshold, but this unavoidably produces a reduction in the performance (Fig. 2). The features of *PER*(*x*) and *PUR*(*x*) depend on the chosen set of parameters. However many sets give rise to functions that are related by simple symmetry transformations like translations or scaling. As long as both functions are transformed in the same way, the purity versus performance curve remains invariant (Fig. 2c). Therefore, parameters {α_i_, μ_i_, σ_i_} are not suitable to classify unequivocally the different samples as they can lead to the same purity-performance curve. In order to find a more appropriate space parameter, we introduce two new dimensionless parameters: the sexual dimorphism index (SDI) and the sexual homoscedasticity index (SHI) defined by

6 $$ SDI=\frac{\mu_f-{\mu}_m}{2\sqrt{\sigma_m{\sigma}_f}} $$

7 $$ SHI=\sqrt{\frac{\sigma_m}{\sigma_f}} $$

Combining definitions (6) and (7) with Equations 4 and 5, we can rewrite performance and purity functions as

8 $$ {\displaystyle \begin{array}{cc} PER(z)& =\Phi \left(\frac{z+ SDI}{\mathrm{SHI}}\right)\times 100\\ {}& \end{array}} $$

9 $$ PUR(z)\kern0.5em =\frac{\alpha_m\varPhi \left(\frac{z+ SDI}{SHI}\right)}{\alpha_m\varPhi \left(\frac{z+ SDI}{SHI}\right)+\left(1-{\alpha}_m\right)\varPhi \left(\frac{z- SDI}{SHI^{-1}}\right)}\times 100 $$

*z* being a dimensionless variable defined by $z=\frac{x-\left(\frac{\mu_m+{\mu}_f}{2}\right)}{\sqrt{\sigma_m{\sigma}_f}}$

Equations 8 and 9 reveal that *PER*(*z*) and *PUR*(*z*) only depend on three dimensionless parameters: α_m_, SDI and SHI. In other words, given α_m_, all combinations of the original parameters {μ_m_, μ_f,_ σ_m_ , σ_f_} giving the same pair {SDI, SHI} have exactly the same performance and purity functions in terms of the dimensionless variable *z*. Consequently, the dimensionless parameters {SDI, SHI} are more suitable than classical measures of center and spread to classify unequivocally different pupae samples with regard to purity and performance.

### Basic statistics and model fit

The mean and standard deviation was estimated for every batch-sex group dataset, and its deviation from the normal distribution was tested by means of the Shapiro-Wilk test. The fit of the data to the probability density function of the mixture model was tested by means of the Kolmogornov-Smirnov test. All the statistic computations were done using the *base* package of R [51]. The significance level was set to α = 0.05.

### Partitioning of the variance

A number of pupae equal to the minimal sample size was randomly selected for each batch-sex group per species/strain. This was performed in order to get a balanced factorial design dataset. A linear model was fit for each batch by means of ordinary least squares. The model included sex and batch as fixed factors, and their interaction. The partitioning of variance was assessed through ANOVA. All statistical analyses were done using the *base* package of R [51].

## Results

Table 1 summarizes the statistics of the pupal size of the species and strains used in the present study. In total, 7733 pupae were analyzed. The majority of the batches were not significantly different from a normal distribution, and the mixture of two Gaussian distributions fitted well to all batches of all the species/strains used when male and female pupae data are combined. Table 2 presents the significance of the goodness-of-fit between the models and the data. None of the samples was significantly different from a Gaussian mixture distribution. Only two batches separated by sex showed significant departures from normality: *An. arabiensis* females of the batch 3, and *Ae. albopictus* Rimini males of batch 3.

**Table 1:** Descriptive statistics for the batches of pupae

Batch	Sex	*N*	Proportion ± SE			Mean size ± SE			SD ± SE		
*Anopheles arabiensis* - Dongola											
1	Males	24	0.48	±	0.10	13.23	±	0.21	1.02	±	0.15
1	Females	26	0.52	±	0.10	14.40	±	0.19	0.99	±	0.14
2	Males	24	0.53	±	0.10	13.23	±	0.20	0.98	±	0.15
2	Females	21	0.47	±	0.11	13.41	±	0.23	1.03	±	0.16
3	Males	32	0.46	±	0.09	13.98	±	0.18	1.03	±	0.13
3	Females	38	0.54	±	0.08	14.16	±	0.16	0.98	±	0.11
Total	Males	80	0.48	±	0.06	13.53	±	0.12	1.07	±	0.08
Total	Females	85	0.52	±	0.05	14.05	±	0.11	1.06	±	0.08
*Aedes albopictus* - Rimini											
1	Males	337	0.53	±	0.03	18.66	±	0.05	0.85	±	0.03
1	Females	303	0.47	±	0.03	21.9	±	0.05	0.87	±	0.04
2	Males	407	0.60	±	0.02	17.73	±	0.04	0.72	±	0.03
2	Females	277	0.40	±	0.03	21.26	±	0.05	0.78	±	0.03
3	Males	434	0.62	±	0.02	18.46	±	0.04	0.84	±	0.03
3	Females	261	0.38	±	0.03	21.62	±	0.05	0.78	±	0.03
Total	Males	1178	0.58	±	0.01	18.26	±	0.03	0.90	±	0.02
Total	Females	841	0.42	±	0.02	21.6	±	0.03	0.85	±	0.02
*Aedes polynesiensis* - Aito (BC9)											
1	Males	164	0.41	±	0.04	17.13	±	0.05	0.66	±	0.04
1	Females	239	0.59	±	0.03	21.09	±	0.05	0.72	±	0.03
2	Males	202	0.49	±	0.04	16.03	±	0.05	0.72	±	0.04
2	Females	210	0.51	±	0.03	19.84	±	0.05	0.69	±	0.03
3	Males	195	0.48	±	0.04	17.03	±	0.04	0.56	±	0.03
3	Females	214	0.52	±	0.03	20.72	±	0.05	0.67	±	0.03
Total	Males	561	0.46	±	0.02	16.70	±	0.03	0.82	±	0.02
Total	Females	663	0.54	±	0.02	20.57	±	0.03	0.87	±	0.02
*Aedes aegypti* - GSS											
1	Males	246	0.47	±	0.03	17.34	±	0.05	0.76	±	0.03
1	Females	272	0.53	±	0.03	20.31	±	0.04	0.73	±	0.03
2	Males	117	0.51	±	0.05	18.20	±	0.07	0.75	±	0.05
2	Females	113	0.49	±	0.05	22.16	±	0.07	0.75	±	0.05
3	Males	496	0.44	±	0.02	18.04	±	0.03	0.72	±	0.02
3	Females	635	0.56	±	0.02	22.24	±	0.04	0.95	±	0.03
Total	Males	859	0.46	±	0.02	17.86	±	0.03	0.81	±	0.02
Total	Females	1020	0.54	±	0.02	21.72	±	0.04	1.22	±	0.03
*Aedes aegypti* - Sri Lanka											
1	Males	314	0.56	±	0.03	16.41	±	0.06	1.08	±	0.04
1	Females	245	0.44	±	0.03	19.58	±	0.08	1.29	±	0.06
2	Males	309	0.55	±	0.03	16.36	±	0.06	0.99	±	0.04
2	Females	249	0.45	±	0.03	20.01	±	0.08	1.26	±	0.06
3	Males	153	0.5	±	0.04	16.36	±	0.08	0.98	±	0.06
3	Females	156	0.5	±	0.04	19.85	±	0.09	1.13	±	0.06
Total	Males	776	0.54	±	0.02	16.38	±	0.04	1.03	±	0.03
Total	Females	650	0.46	±	0.02	19.81	±	0.05	1.25	±	0.03
*Aedes aegypti* - WB2-BRA											
1	Males	270	0.68	±	0.03	17.65	±	0.04	0.70	±	0.03
1	Females	129	0.32	±	0.04	21.23	±	0.07	0.83	±	0.05
2	Males	86	0.44	±	0.05	18.39	±	0.07	0.66	±	0.05
2	Females	111	0.56	±	0.05	22.37	±	0.09	0.99	±	0.07
3	Males	160	0.38	±	0.04	19.12	±	0.06	0.74	±	0.04
3	Females	264	0.62	±	0.03	23.15	±	0.06	0.95	±	0.04
Total	Males	516	0.51	±	0.02	18.23	±	0.04	0.96	±	0.03
Total	Females	504	0.49	±	0.02	22.48	±	0.05	1.22	±	0.04

Batch, number of the batch, corresponding to a rearing container; *N*, number of pupae; Proportion, proportion of pupae; Mean size (√pixel) for the group; SD, Standard deviation; SE, standard error

**Table 2:** Results for the goodness-of-fit of the data to a probability distribution function. *P*-values are provided for each test

Species/Strain	Batch	S-W		K-S
		Males	Females	Mixture
		*P*-value	*P*-value	*P*-value
*An. arabiensis* Dongola	1	0.7334	0.7329	0.9094
	2	0.7290	0.2108	0.7998
	3	0.9345	0.0102*	0.4434
*Ae. albopictus* Rimini	1	0.1545	0.3109	0.9730
	2	0.0526	0.6420	0.6992
	3	0.0021*	0.3856	0.8665
*Ae. polynesiensis* (Aito (BC9)	1	0.3576	0.0488	0.9371
	2	0.7452	0.3139	0.9556
	3	0.6154	0.5026	0.7538
*Ae. aegypti* GSS	1	0.6916	0.2393	0.7966
	2	0.1250	0.9713	0.9872
	3	0.4354	0.7882	0.9726
*Ae. aegypti* Sri Lanka	1	0.4785	0.5730	0.9305
	2	0.6273	0.8647	0.976
	3	0.7664	0.3403	0.9897
*Ae. aegypti* WB2-BRA	1	0.1064	0.1099	0.7892
	2	0.3019	0.1325	0.9840
	3	0.6914	0.0480	0.5103

*Abbreviations:* S-W, Shapiro-Wilk test for the test of normality of the distributions of each sex; K-S, Kolmogorov-Smirnov test for the fit to a mixture of normal distributions

**Fig. 3:**
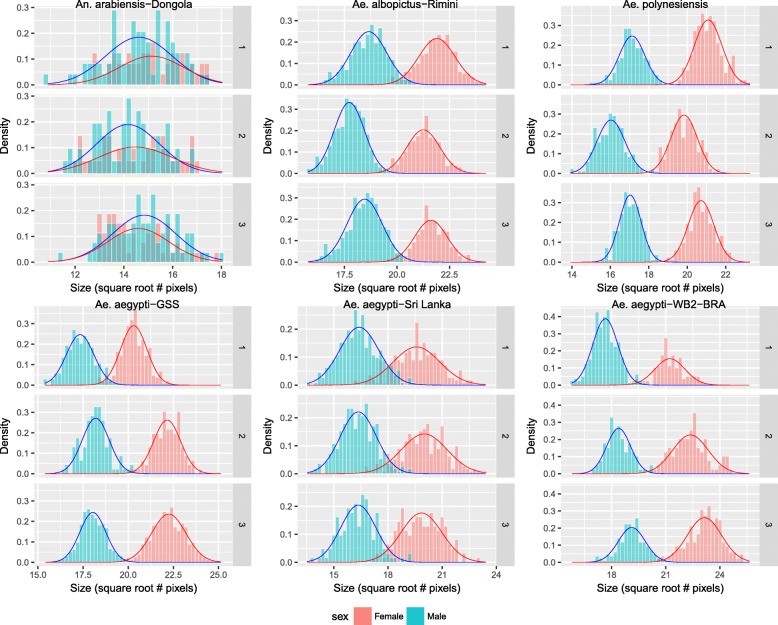
Frequency distribution of size (√pixel) for pupae of different mosquito species and strains reared under small-scale laboratory conditions. Males are represented in blue and females in red. Three replicates are presented for each mosquito species/strain

**Fig. 4:**
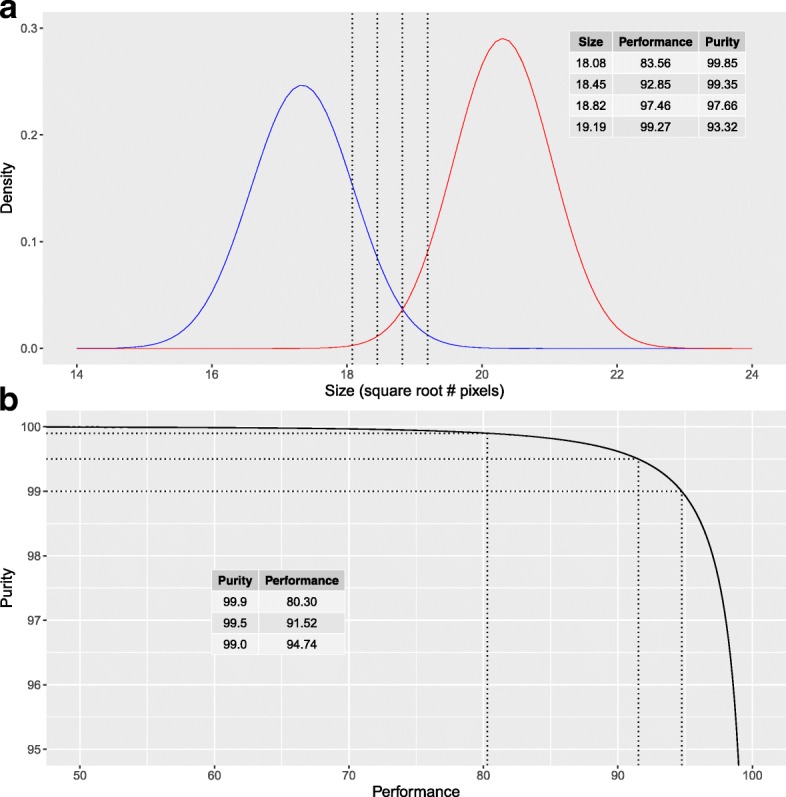
Depiction of the SSD-sorting methods functioning simulated by a mixture of two normal distribution functions. Different threshold of sizes separates the sample in two subsamples. The subsample of smaller size has a different male proportion depending on the chosen threshold. Purity = % males on the sorted sample. Performance = % males recovery. The dotted lines depict a value of threshold. **a** Probability density function of male and female pupal size. **b** Purity *versus* performance

Figure 3 shows the histograms and fitted models for the three batches of each species/strain studied. After the parameters of the model have been estimated, the purity-performance characteristic curve is computed and the quality of sorting can be analyzed theoretically. Each value of pupal size is linked to a pair of purity and performance values, which are inversely related (Fig. 4). The fitted models allow simulating the output of SSD-sorting methods under different circumstances. Table 3 shows the main descriptors for the predicted output from the fitted model for each batch in the experimental data. These results should not be considered as a general prediction of how a particular strain will perform with SSD-sorting methods, since they are only applicable for the specific rearing conditions of this experiment. However, they show the potential of SSD-based sex-sorting procedures when standard rearing procedures are applied. Table 4 provides examples for one of the strains of *Ae. aegypti* (GSS) of how the performance-purity output varies under different simulated conditions. The simulation *a* describes the performance when the three batches are mixed and a threshold size is determined by keeping constant the level of purity of 99.5 %. It is shown how SDI takes lower values than any of the individual batches, and an average reduction of 17 % in performance is predicted for the same level of purity (male recovery of 74.5 % with a female contamination of 0.5 % after mixing the three batches). Simulations *b* and *c* assess the effect in the performance of the size heterogeneity and the variations in the SSD respectively. For simulation *b* the variance of both sexes is scaled by the same factor while the distance between means remains constant. Taking batch 1 as a reference, the standard deviation of both sexes is multiplied by 0.8 and 1.2 respectively. For simulation *c*, taking again batch 1 as reference, the average size of males and females is increased or decreased by 0.5 √pixels but the standard deviations are not modified in this case. It is worth mentioning that the variation of the statistical parameters in simulations *b* and *c* only affect the dimensionless parameter SDI, while SHI remains unaltered. Results show that changes in size heterogeneity and SSD have contrary effects in the performance; an increase in intra-batch variance produces a significant drop in performance whereas an increase in SSD improves the quality of sorting. Simulation *d* describes how the output of SSD-sorting systems vary with the election of a predefined constant threshold for all the batches.

**Table 3:** Predictions of the fitted models with the experimental parameters for different measures of suitability to sexual size dimorphism sorting methods. Performance (% males recovery) at different levels of purity (% males in the sorted sample)

Species/Strain	Batch	SDI	SHI	Performance		
				Purity = 99.9	Purity = 99.5	Purity = 99.0
*An. arabiensis* Dongola	1	0.58	1.01	<0.01	0.04	0.23
	2	0.09	0.98	-	-	-
	3	0.09	1.02	<0.01	<0.01	-
	Total	0.24	1.00	<0.01	<0.01	-
*Ae. albopictus* Rimini	1	1.89	0.99	73.09	88.45	92.96
	2	2.35	0.96	95.12	98.78	99.45
	3	1.95	1.04	83.92	93.55	96.19
	Total	1.91	1.02	78.97	91.13	94.62
*Ae. polynesiensis* Aito (BC9)	1	2.87	0.96	99.41	99.89	99.96
	2	2.69	1.03	98.93	99.74	99.88
	3	3.00	0.92	99.78	99.97	99.99
	Total	2.29	0.97	91.41	97.25	98.55
*Ae. aegypti* GSS	1	2.00	1.02	80.28	91.51	94.74
	2	2.65	1.00	98.65	99.68	99.86
	3	2.54	0.87	95.17	99.03	99.61
	Total	1.94	0.81	33.75	74.51	86.43
*Ae. aegypti* Sri Lanka	1	1.35	0.92	5.54	31.16	48.47
	2	1.63	0.89	24.63	60.80	75.26
	3	1.67	0.93	37.38	67.23	78.52
	Total	1.51	0.90	15.66	48.85	64.99
*Ae. aegypti* WB2-BRA	1	2.35	0.92	95.69	99.15	99.67
	2	2.46	0.82	89.46	97.94	99.21
	3	2.40	0.89	89.61	97.23	98.69
	Total	1.96	0.89	63.59	86.67	92.77

*Abbreviations*: SDI, SHI, dimensionless parameters, characteristic of each batch

**Table 4:** Predicted values for the descriptive parameters of the sex sorting of the *Ae. aegypti* GSS strain simulated under different conditions

Sim.	Conditions	Group	μ_m_	μ_f_	σ_m_	σ_f_	SDI	SHI	Performance	Purity
-	Experimental	Batch1	17.34	20.31	0.76	0.73	2.00	1.02	91.51	99.5
		Batch 2	18.20	22.16	0.75	0.75	2.65	1.00	99.68	99.5
		Batch 3	18.04	22.24	0.95	0.72	2.54	0.87	99.03	99.5
*a*	Combination of batches	Total	**17.86**	**21.72**	**1.22**	**0.81**	1.94	0.81	74.51	99.5
*b*	Differences in	Batch 1	17.34	20.31	0.76	0.73	2.00	1.02	91.51	99.5
	intra-batch variance.	sd* 1.2	17.34	20.31	**0.91**	**0.87**	1.66	1.02	74.38	99.5
	SSD constant	sd* 0.8	17.34	20.31	**0.61**	**0.58**	2.49	1.02	99.12	99.5
*c*	Differences in SSD	Batch 1	17.34	20.31	0.76	0.73	2.00	1.02	91.51	99.5
	intra-batch. Variance,	SSD-0.5	**17.59**	**20.06**	0.76	0.73	1.66	1.02	74.14	99.5
	Constant	SSD+0.5	**17.09**	**20.56**	0.76	0.73	2.33	1.02	97.99	99.5
*d*	Fixed threshold	Batch1	17.34	20.31	0.76	0.73	2.00	1.02	93.69	99.22
	**x=18.5** √pixels	Batch 2	18.20	22.16	0.75	0.75	2.65	1.00	65.54	100.00
		Batch 3	18.04	22.24	0.95	0.72	2.54	0.87	73.80	99.99

*Abbreviations*: Sim, reference for the simulation; Group, specific simulation for the group; μ_m_, μ_f_, σ_m_, σ_f_, parameters of the model; SDI, SHI, dimensionless parameters; Performance, % males recovery; Purity, % males on the sorted sample

Simulations: *a*, effects of sex-sorting after combining the three batches; *b*, effects of increase/decrease the variance; *c*, effects of increase/decrease the difference in means between sexes; *d*, effects of sex-sorting three batches with a predefined size threshold. For each simulation, the parameters that are modified from the experimental data (first 3 rows) are highlighted in bold style

The contribution of different factors to the variability in size has been assessed by means of ANOVA. The results for the partitioning of the variance are shown in Table 5. For all the *Aedes* species, the biggest source of variation is the SSD. For *An. arabiensis*, there are significant differences in size between sexes, but this factor explained only a relatively small portion of the total variance.

**Table 5:** ANOVA tables for each species/strain. The factors included are batch (rearing container) and sex (male or female)

Species/Strain		df	SS	MS	*F*	*P*	
*An. arabiensis* Dongola	Batch	2	9.6	4.798	5.298	0.006	***
	Sex	1	6.81	6.807	7.515	0.007	***
	Batch:Sex	2	10.29	5.145	5.681	0.004	***
	Residuals	120	108.69	0.906			
*Ae. albopictus* Rimini	Batch	2	174	87	133.646	<0.001	***
	Sex	1	4281	4281	6565.183	<0.001	***
	Batch:Sex	2	10	5	7.864	<0.001	***
	Residuals	1560	1017	1			
*Ae. polynesiensis* Aito (BC9)	Batch	2	263	131	295.618	<0.001	***
	Sex	1	3625	3625	8155.481	<0.001	***
	Batch:Sex	2	4	2	4.289	0.014	*
	Residuals	978	435	0			
*Ae. aegypti* GSS	Batch	2	278.2	139.1	223.99	<0.001	***
	Sex	1	2306.3	2306.3	3713.93	<0.001	***
	Batch:Sex	2	32.9	16.4	26.45	<0.001	***
	Residuals	672	417.3	0.6			
*Ae. aegypti* Sri Lanka	Batch	2	11.8	5.9	4.587	0.0104	*
	Sex	1	2764.1	2764.1	2152.107	<0.001	***
	Batch:Sex	2	10	5	3.892	0.020	*
	Residuals	912	1171.4	1.3			
*Ae. aegypti* WB2-BRA	Batch	2	260.3	130.2	208.85	<0.001	***
	Sex	1	1926.1	1926.1	3090.347	<0.001	***
	Batch:Sex	2	8.3	4.1	6.627	0.001	**
	Residuals	510	317.9	0.6			

*Abbreviations*: df, degrees of freedom; SS, sum of squares; MS, mean sum of squares; *F*, *F*-statistics; *P*, *P*-value for the *F*-statistics

## Discussion

For all the species and strains examined in this study, the joint frequency distribution of pupal size included an area of overlap between the individual male and female distributions. This essentially means that a complete separation of sexes according to a given threshold of size is not possible, and every threshold that separates the sample in two will leave a certain proportion of each sex in the batch of the other group: smaller females in the male group and/or bigger males in the female group. This limitation of the SSD-based sorting methods is commonly recognized, altogether with the general observation that rearing conditions have a strong effect over the performance of the methods [8, 20–22, 52]. However, the mechanisms under these observations have not been investigated in depth, which may affect the optimization of new sorting methods based on SSD.

For the four species analyzed, including the three *Ae. aegypti* laboratory strains, the distribution of the size of each sex considered apart followed a normal distribution, as commonly observed in insects [33]. For the three *Aedes* species studied here, the joint frequency distribution for both sexes is noticeably bimodal, and can be modeled through a mixture of two normal probability density functions. This model is rather simple, with only five parameters that can be easily estimated directly from a population sample. It is likely that this approach can be generalized to other *Aedes* species as well as to culicine mosquitoes with a marked dimorphism in size [31, 38, 53]. In addition, this method of analysis could be generally applied to all known SSD-based sorting methods, since all of them rely on separating batches of pupae in two groups through the definition of a threshold size.

The features of the distribution in sizes of the individuals determine the differences between samples/ strains/ species in the suitability for any SSD-sorting method. These differences are reflected in two main parameters: the performance (% males recovery) and sample purity (% males on the sorted sample). Both can be predicted from the probability density function. For a given set of the model parameters (α_m_; μ_i_, σ_i_), each size threshold has a pair of values of performance and purity associated. Under these model assumptions, purity and performance in each sample of pupae are unequivocally linked; each value of purity corresponds to a single value of performance. Since the evaluation of the quality of a given sorting through the predicted values of purity-performance depends on the chosen value of threshold, the relationship of both parameters in a dimensionless space has been analyzed theoretically. This analysis has provided two useful indices that describe the applicability of SSD-sorting methods for a given sample of pupae, i.e. the quality of the biological material and the rearing conditions. As SDI increases, the purity-performance function becomes more optimal (better performance with higher purity). The SDI index has two components, the SSD and the sample variance. Consequently, an increase in SSD and a reduction in variance increase the efficiency of any SSD-sorting method, as will be discussed later. Index SHI modifies the slope of the curve. The higher the SHI value, the more flattened purity-performance curve is obtained. This parameter describes the difference in variance between males and females, which is difficult to control during the rearing process. SDI and SHI can be used for long-term monitoring of the quality control of the sorting process.

The purity and performance are inversely correlated. From the applied point of view, any sorting system must choose a size threshold considering the trade-off between performance and purity. From Equations 8 and 9, it is possible to estimate, for a given sample, the threshold of size needed to obtain a desired value of purity or performance. Unfortunately, it is not possible to calculate a single constant size threshold for sex sorting that keeps constant the purity and performance levels across different batches. It is known that there is heterogeneity in size in the production units (rearing containers) that affects the outcome of the sorting methods [8, 20, 21, 52]. For instance, the three *Ae. aegypti* GSS SSD batches varied in purity (99.2-100%) and performance (66-94%) when separated by the same threshold value (see Table 4).

Keeping the purity as a constant parameter, and assuming heterogeneity in size, the outcome of the sorting will have a variable percent recovery of males. Since this heterogeneity is important in the output of the sex sorting, we analyzed and quantified the sources of variation in size in the experimental sample. Then, we used the parameters directly estimated from the samples to predict the expected values of purity and performance for each batch of pupae. Changing the value of these parameters in the fitted models allowed us to simulate the outcome of SSD-sorting methods under different scenarios. The partitioning of variance showed two different patterns of relative importance in respect to the source of heterogeneity in size. For the *Aedes* species, the main source of variation was the sexual difference, followed by the residual, the differences between batches and finally the interaction sex-batch. For *Anopheles*, the effect of sex was of less importance, and the residual accounted for most of the variation.

The SSD, as the absolute difference between mean size of each sex, is the main factor that explains the interspecific differences in the applicability of SSD-sorting methods. A higher SSD will produce higher performance independently of the scale, and for all the size thresholds considered, yielding a higher SDI. The two species with higher SSD (*Ae. aegypti* and *Ae. polynesiensis*) are known to yield better results than *Ae. albopictus* when separated with plate separators. On the other hand, *An. arabiensis,* as expected [11, 12], showed a poor suitability for the SSD-sorting methods. The experimental samples of *An. arabiensis* showed an average SSD of 0.52 √pixels, while the *Aedes* species ranged from 3.3 to 3.9 √pixels. Likely, even achieving a reduction in the heterogeneity would not be enough to make SSD-sorting methods suitable for *An. arabiensis* or related species. For example, a SSD-sorting method was used to sort *An. albimanus* in a trial in El Salvador [54], and resulted in 14% of female contamination in the released mosquitoes which would be currently unacceptable.

The residual variance is the second important source of variation in the *Aedes* group. It accounts for the unexplained variation due to other factors, mainly the natural heterogeneity in size that can be found in any pupal batch. The heterogeneity in size in a given batch (rearing container) has a strong effect on the performance. As an example, our simulations (Table 4) with the GSS strain predict that a 20 % increase in the standard deviation of the experimental value reduces the performance by about 17 %. Conversely, a reduction of the same magnitude produces an increase in male recovery of 7.6 %. The heterogeneity in size could be due to genetic and/or environmental factors [55, 56]. It is not expected that the genetic heterogeneity of laboratory populations has a major effect given that it has been drastically reduced by the colonization process [55]. On the other hand, it is known that intraspecific asymmetric density-dependent factors can increase the variability in size in other insects which have an aquatic larval developmental stage [57, 58]. Given that the mosquito larvae are usually kept at high densities in the artificial rearing containers, the heterogeneity in size could potentially be reduced by adjusting the larval density.

The variance between rearing containers is also an important factor to consider in SSD-sorting. In a real mass production context, there is variation in size between batches that is present in our experiments as well. This variation affected mainly the average size of the pupae, but also at some extent the absolute SSD magnitude (Table 5, Interaction term Batch:Sex). It has been reported that the food availability or the water pollution by conspecifics does not affect the absolute SSD magnitude in *Aedes* [38, 43] while larval competition could produce some degree of sexual allometry in size [40, 42]. The intraspecific variation in SSD is a complex issue [35–37] out of the scope of this article, but worth to be investigated in mosquitoes in the context of SSD-sorting methods. The variability between rearing containers is of major applied significance because the threshold of size needed to obtain a desired degree of purity is specific for each batch. The use of a common fixed threshold for all the production batches would produce a variable output in respect to purity and performance, and it is therefore not recommended (Table 4). In a mass production context, it can be sometimes useful to mix the pupae production of different rearing containers before the sex sorting, but this would likely increase the size heterogeneity. This is clearly shown in the simulations presented in Tables 3 and 4. For example, mixing the *Ae. aegypti* GSS pupae production from three rearing trays reduced the performance in about 17 % (with purity level of 99.5 %).

Two main strategies are presently used for SSD sorting methods. First, sieves [8], rows of slots [21] or openings between plates [22], which are based on fixed size thresholds, were developed through a trial and error process. This means that the purity and performance are not controlled and they entirely depend on the rearing conditions. On the other hand, plate separators [23], which rely on a visual adaptive size threshold election system, exhibit better performance [52] at the expense of productivity [21]. Both strategies can be optimized using the appropriate analytical tool. For the fixed threshold methods, a more optimal threshold based on the actual range of variation in size between batches of pupae may be required. The plate separator could be optimized by the determination of less subjective threshold election criteria. Finally, the analytical framework proposed here can be integrated in large scale mechanized sex-sorters of high precision.

## Conclusions

The distribution of size in mosquito pupae can be modeled by a mixture of two Gaussian distribution functions. This approach, combined with the parameters obtained from laboratory samples, can be useful to understand and optimize the mechanisms of the SSD-sorting methods. Purity and performance, which are the most relevant features of sex sorting devices, can be directly calculated from the presented model. Two additional dimensionless parameters, SDI and SHI, which are good descriptors of the suitability of a species/strain under given rearing conditions for its sorting with SSD-based methods are proposed. This approach can be applied to all the SSD-sorting methods. The output of the SSD-sorting methods can be improved by reducing the heterogeneity in size within the rearing containers. The heterogeneity between batches can affect the quality of sex sorting when different batches are mixed before the sorting or when a common separation threshold is determined for a series of batches. For new designs of sex-sorting devices based on SSD, we recommend the following: (i) use of an adaptive and precise threshold selection method based on automatic measurement systems and the proposed formulas; and (ii) a specific threshold size for each batch to maintain the purity at a constant level. In this way, the heterogeneity in size will be resulting to a variable male recovery (performance). From the practical point of view, this study shows that enhanced SSD-based sex sorting methods can be applied to *Aedes* mosquito mass-rearing facilities that depend on lateral area to distinguish sexes to efficiently produce batches of male-only pupae with a male recovery ranging between 70% and 99% and female contamination under 0.5%, with the lower values of male recovery being obtained when different batches are mixed or when larval rearing conditions are not standardized.

## Abbreviations

GSS: Genetic sexing strain
IIT: Incompatible insect technique
RIDL: Release of insects carrying a dominant lethal
SDI: Sexual dimorphism index
SHI: Sexual homoscedasticity index
SIT: Sterile insect technique
SSD: Sexual size dimorphism
